# Advances in CAR T cell therapy: antigen selection, modifications, and current trials for solid tumors

**DOI:** 10.3389/fimmu.2024.1489827

**Published:** 2025-01-06

**Authors:** Safwaan H. Khan, Yeonjoo Choi, Mysore Veena, John K. Lee, Daniel Sanghoon Shin

**Affiliations:** ^1^ Department of Medicine, Division of Hematology/Oncology, David Geffen School of Medicine at University of California, Los Angeles (UCLA), Los Angeles, CA, United States; ^2^ Department of Microbiology, Immunology, and Molecular Genetics, University of California, Los Angeles (UCLA), Los Angeles, CA, United States; ^3^ Division of Hematology/Oncology, Veterans Affairs (VA) Greater Los Angeles Healthcare System, Los Angeles, CA, United States; ^4^ Department of Molecular and Medical Pharmacology, University of California, Los Angeles, Los Angeles, CA, United States

**Keywords:** CAR T cell, solid tumors, immunotherapy, cancer, structure, targets

## Abstract

Chimeric antigen receptor (CAR) T cell therapy has revolutionized the treatment of hematologic malignancies, achieving remarkable clinical success with FDA-approved therapies targeting CD19 and BCMA. However, the extension of these successes to solid tumors remains limited due to several intrinsic challenges, including antigen heterogeneity and immunosuppressive tumor microenvironments. In this review, we provide a comprehensive overview of recent advances in CAR T cell therapy aimed at overcoming these obstacles. We discuss the importance of antigen identification by emphasizing the identification of tumor-specific and tumor-associated antigens and the development of CAR T therapies targeting these antigens. Furthermore, we highlight key structural innovations, including cytokine-armored CARs, protease-regulated CARs, and CARs engineered with chemokine receptors, to enhance tumor infiltration and activity within the immunosuppressive microenvironment. Additionally, novel manufacturing approaches, such as the Sleeping Beauty transposon system, mRNA-based CAR transfection, and *in vivo* CAR T cell production, are discussed as scalable solution to improve the accessibility of CAR T cell therapies. Finally, we address critical therapeutic limitations, including cytokine release syndrome (CRS), immune effector cell-associated neurotoxicity syndrome (ICANS), and suboptimal persistence of CAR T cells. An examination of emerging strategies for countering these limitations reveals that CRISPR-Cas9-mediated genetic modifications and combination therapies utilizing checkpoint inhibitors can improve CAR T cell functionality and durability. By integrating insights from preclinical models, clinical trials, and innovative engineering approaches, this review addresses advances in CAR T cell therapies and their performance in solid tumors.

## 1 Introduction

Adoptive cellular therapy (ACT), pioneered by Steven Rosenberg, utilizes the engineering of one’s own immune system to fight against malignancies. Chimeric Antigen Therapy (CAR T) is a form of ACT that has had tremendous impact in cancer therapy. The first approved CAR T therapy dates to 2017, when it was used for the treatment of hematological malignancies.

CAR T therapy utilizes the engineering of T cells to fight diseases in patients. T cells can be engineered to have heightened sensitivity towards target antigen – these T cells can then identify and begin an immune response against cells presenting the target antigen (1). While the basic structure of the CAR is well defined with an antigen binding domain, a transmembrane domain, and an intracellular signaling domain, there have been evolutions in the structures of CARs to allow for better immune response and greater antigen sensitivity (2). These advancements are fundamental for the potential expanded use of CAR T therapy.

The successes of CAR T therapy in hematological malignancies have opened doors for the utilization of this therapy to treat various forms of cancers, including solid tumors. Since CAR T therapy’s inception, its target range has considerably grown. The approach offered by CAR T therapy provides a unique advantage given that an engineered T cell can target any surface antigen whereas endogenous T cells can only recognize antigens presented on a major histocompatibility complex (MHC). This growth in target range comes with its own challenges. Namely, one of the primary challenges with CAR T therapy is the identification of truly tumor specific or tumor associated antigens. Thus, one area of research has been antigen identification for the development of specific and effective CAR T therapies (3).

Several CAR T cell products have been approved by the U.S. Food and Drug Administration (FDA) and are being integrated into treatment regimens for hematologic malignancies. These include CD-19 targeting therapies, such as tisagenlecleucel (Kymriah), axicabtagene ciloleucel (Yescarta), brexucabtagene autoleucel (Tecartus), and lisocabtagene maraleucel (Breyanzi), which have demonstrated exceptional efficacy in treating relapsed or refractory B-cell lymphomas and acute lymphoblastic leukemia (ALL) (4–7). Additionally, idecabtagene vicleucel (Abecma) and ciltacabtagene autoleucel (Carvykti), targeting B-cell maturation antigen (BCMA), have shown significant clinical benefits in patients with multiple myeloma (8–10). These therapies, which were initially used in relapsed or refractory setting, are now being explored in earlier lines of treatment, underscoring their potential to become first-line options for certain hematologic cancers.

Currently, CAR Ts are being explored for patients with various advanced solid tumors. However, CAR T therapies for treatment of solid tumors have yet to display their potential. The progress is limited due to a plethora of challenges – tumor accessibility, antigen heterogeneity, lack of specific tumor associated antigens (TSA) compared with hematologic malignancies, on target off tumor toxicity, cytokine release syndrome, neurological toxicities, the immunosuppressive properties of the tumor microenvironment, and T cell dysfunction driven by chronic antigen exposure (11–13). Rigorous studies on these areas of concern have led to promising translatable results that could soon cement CAR T therapy as one of the most effective treatment options for solid tumors. Approaches to address these areas of concern include modifications to the antigen binding domains of CARs, new methods to discover more cancer-enhanced antigens, enhancements to CAR T structure to allow for better solid tumor infiltration, and gene editing approaches to increase CAR T cell activity (14–20).

Additionally, the ability to produce CAR T cells at large using allogeneic T cells would allow for more widespread applications of CAR T therapies. While this approach does increase the risk for graft versus host disease, advancements in the methodologies used to produce allogeneic T cells have significantly lowered the adverse effects of such an approach. Thus, the use of allogeneic T cells allows for greater accessibility of CAR T therapy in its “off-the-shelf” form as compared to autologous T cell-based CAR T therapy.

The primary objective of this literature review is to specifically address the advances in CAR T cell therapy, namely in tumor-specific antigen identification and CAR modifications, and the performance of these advances in CAR T cell therapy clinical trials for solid tumors. Through an overview of CAR T structure, classification, modification, and manufacturing followed by an analysis of antigen selection techniques and clinical trials of CAR T cell therapies against identified antigens, we will discuss the current state of CAR T cell therapies in treating solid tumors while also presenting scopes for improvement for current therapeutic limitations and challenges.

## 2 Overview of CAR structure

**Figure 1:**
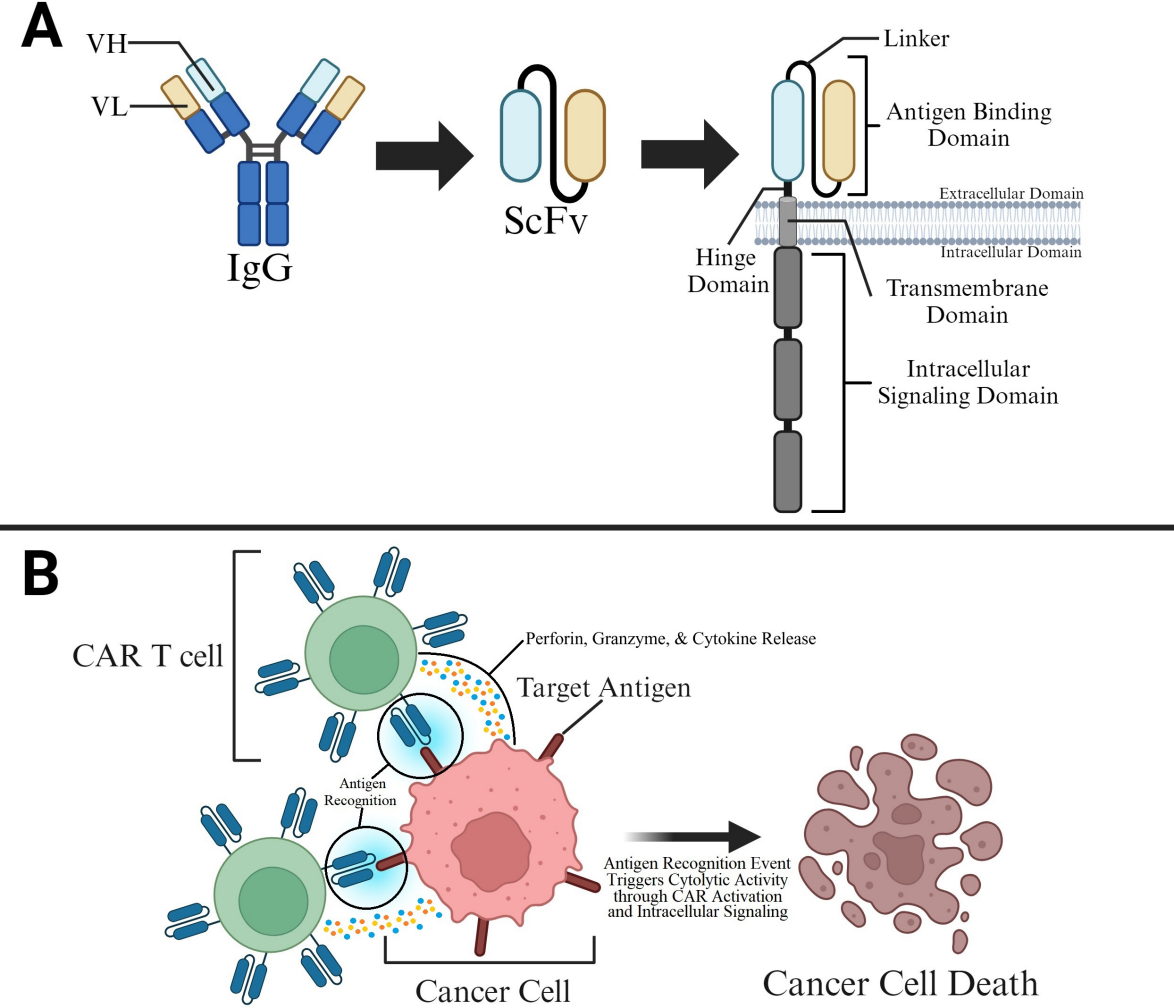
CAR Structure and Function in Killing Cancer Cells **(A)** The CAR’s antigen binding domain is typically derived from a single-chain variable fragment (scFv). The antigen binding domain is present on the ectodomain of the T cell and is connected to hinge, transmembrane, and intracellular signaling domains that allow for CAR stability and binding induced attack of cancer cells by T cells. **(B)** CAR T cell binding to target antigens on cancer cells allows for T cell activation and T cell mediated killing of cancer cells.

CARs are similar to T cell receptors (TCR) in their composition of membrane-bound signaling receptors with ligand-binding domains followed by spacer ectodomains, a transmembrane domain, and one or more cytoplasmic domains (1, 21, 22) (**Figure 1A**). Typically, a CAR consists of four main components: (i) Target antigen-binding domain (extracellular). This is typically derived from the variable heavy (VH) and light (VL) chains of monoclonal antibodies that are bound by a linker to form single-chain variable fragments (scFv); however, another approach for CAR binding relies on variable heavy domains of heavy chains (VHHs). Further specification regarding the utilization of these VHHs, or nanobodies, is discussed in the “Modification of CAR T Cells” section. The target antigen-binding domain is involved in target identification and binding, which confers target specificity to CARs. (ii) Spacer region (also called hinge). This region provides flexibility to the antigen-binding domain, allowing for enhanced recognition of target epitopes. (iii) Transmembrane domain. This domain anchors the extracellular and intracellular domains to the cellular membrane and plays an important role in determining the CAR membrane stability and surface expression. (iv) Signaling domains (intracellular and may vary in number). Signaling domains are involved in downstream signaling after binding of the antigen-binding domain with the target antigen is complete. The intracellular signaling actions of these domains, which will be further discussed in the “CAR T Classification” section, ultimately lead to signaling cascades that activate T cell mediated cancer cell attack.

Designing CARs requires careful evaluation of these several factors. Subsequently, a CAR T cell is generated by packaging these synthetic domains into viral vectors and transducing them into T cells. The flexibility of CAR modular composition provides CAR T cells with great versatility and tunability when used in therapeutic applications. During an antigen encounter event, the antigen-binding domain binds to its target, triggering conformational changes in the entire protein. As a result, several post-translational modifications (PTMs) are initiated that commence intricate intracellular signaling cascades, culminating in T cell activation (**Figure 1B**). Overall, the creation of custom CAR constructs can be achieved through plasmid editing that allows for insertions, alterations, and deletions of domain-encoding sequences.

## 3 CAR T classification

**Figure 2:**
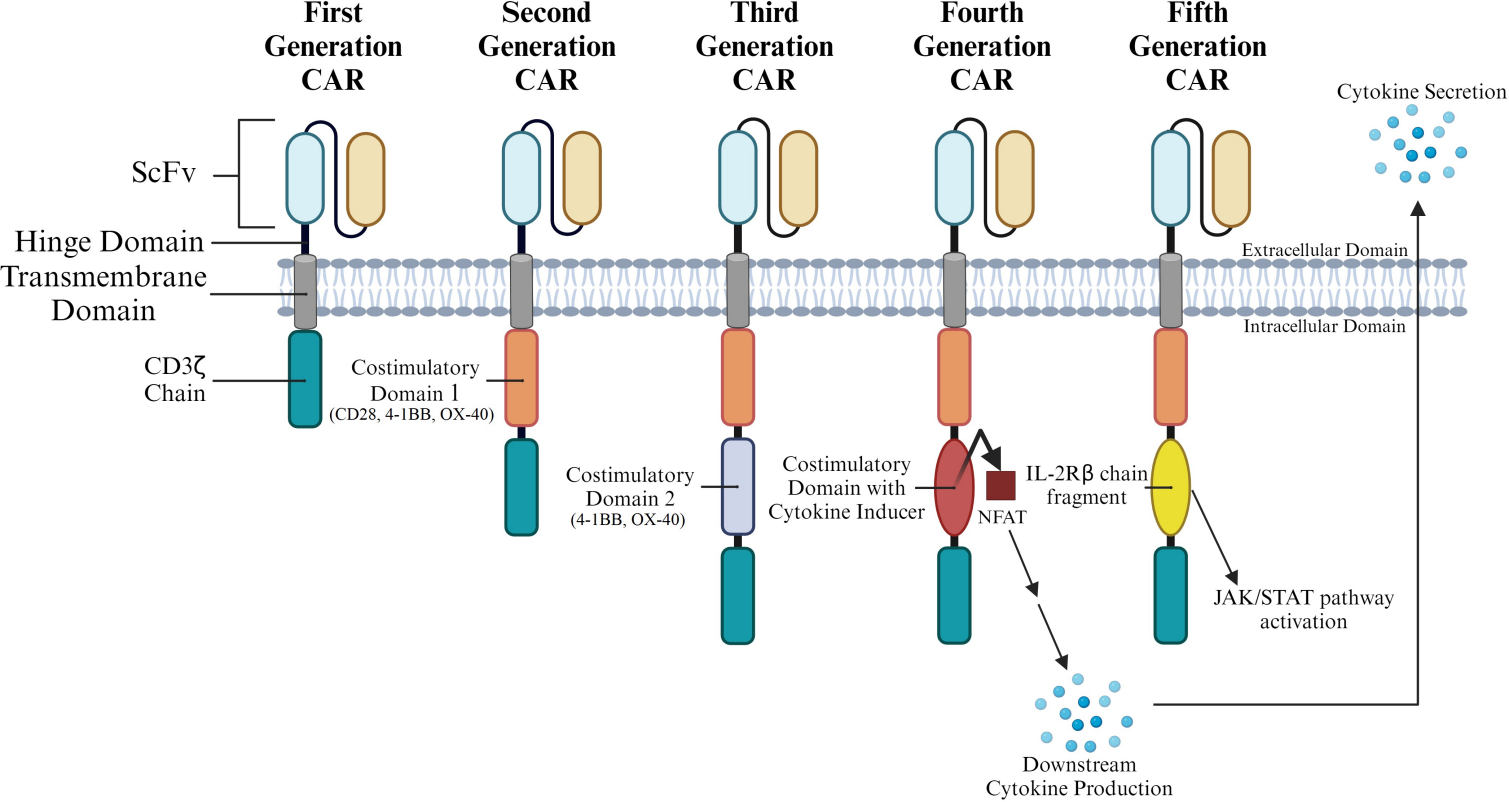
CAR T Generation Classification by Intracellular Structure. CARs are commonly grouped into generations based on the various structures present in their intracytoplasmic domains. Multiple costimulatory domains and combinations of costimulatory domains have been analyzed. Parentheses under costimulatory domains 1 and 2 represent specific domains that have been tested at that position throughout CAR generations.

Currently, CAR T cells are classified into five generations according to the organization of their intracellular signaling domains (**Figure 2**). The intracellular domains are important in inducing differentiation, bringing about cytotoxic response, producing cytokines, and recruiting other immune cells. These events enhance the process of tumor elimination and allow non-MHC restricted targeting of tumors. Hence, current research focus aims to enhance CAR T cell clinical efficacy by amplifying and finetuning these functions.

### 3.1 First generation CARs

First-generation CARs carry a single CD3ζ-chain or FcϵRIγ intracellular domain (**Figure 2**) without any additional costimulatory domains. Though these complexes are like endogenous TCRs, they are unable to produce sufficient interleukin-2 (IL-2). As a result, first-generation CARs need to be supplemented with exogenous IL-2 for an efficient response. This proved to be a major drawback in the first-generation CAR Ts (11). First generation CARs have also demonstrated low cell proliferation and a short *in vivo* lifespan; both these points contributed to their poor performance in a clinical setting (12, 13). These drawbacks and poor clinical performance prompted the development of the second generation of CAR Ts.

### 3.2 Second generation CARs

To improve on the first generation of CARs, co-stimulatory domains such as CD28, 4-1BB, or OX-40 were added to the CAR structure (**Figure 2**). CARs carrying these cytoplasmic costimulatory domains proved much more effective in delivering a secondary signal upon encountering a tumor antigen. A comparison of T cells carrying only one signaling domain to those carrying an internal CD28 costimulatory domain showed a significant increase in proliferation and persistence (23). In general, addition of co-stimulatory domains improved proliferation and cytotoxicity, while also leading to a prolonged response and an increased *in vivo* life span. Each of these costimulatory domains imparted specific characteristics to the CAR T. 4-1BBζ-CAR T cells could persist longer in circulation than CD28ζ-CAR T. It was also observed that while the former caused early exhaustion of CAR T cells, the latter led to constitutive stimulation even in the absence of the antigen. Thus, subsequent CAR design focused on the engineering of more effective costimulatory constructs (23).

### 3.3 Third generation CARs

**Figure 3:**
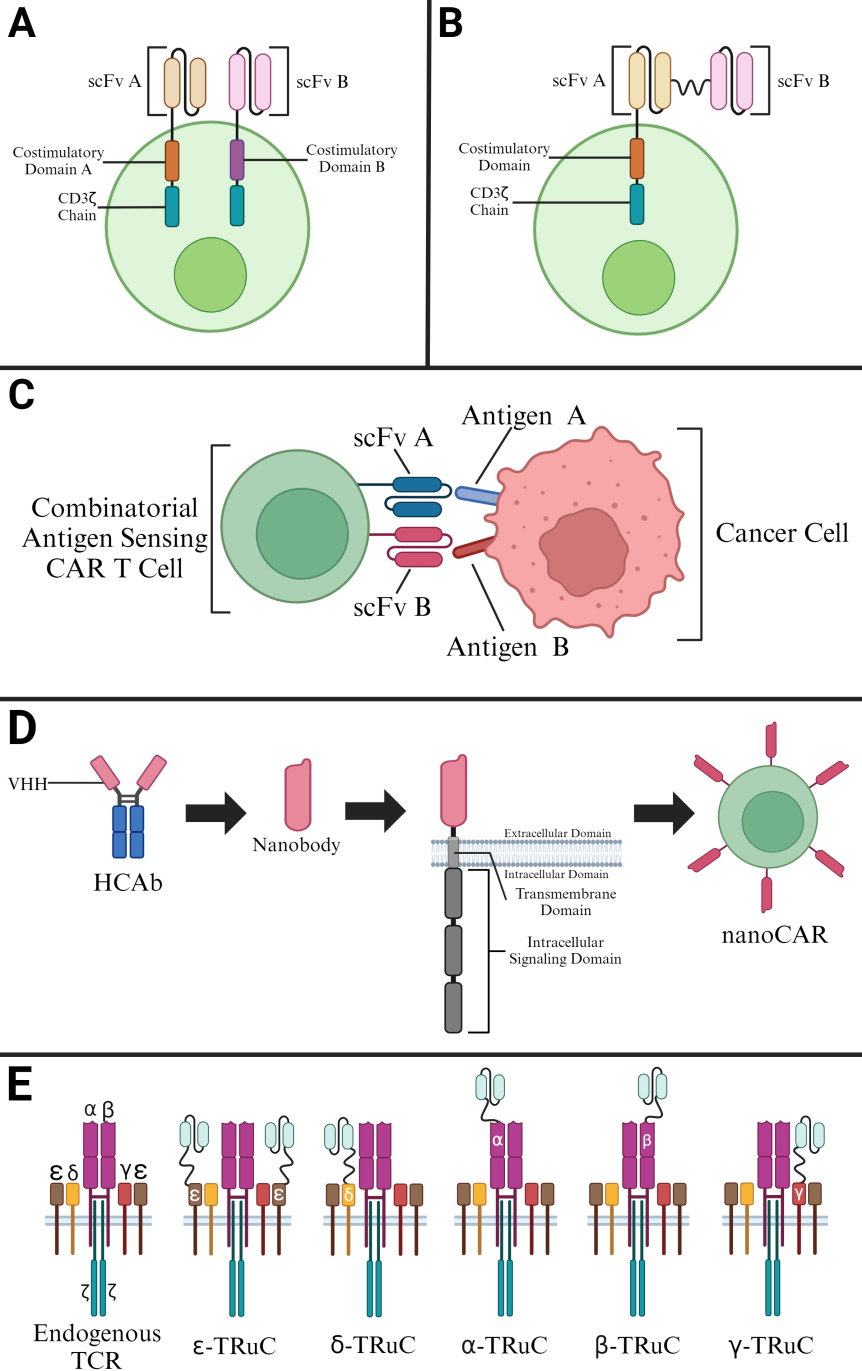
Selected Modifications of CAR T Cells. **(A)** Construction of a dual-signaling CAR utilizing a second-generation CAR. **(B)** Construction of a tandem CAR utilizing two scFvs in a second-generation CAR. **(C)** Construction of a CAR with Combinatorial Antigen Sensing Circuits in the form of an “AND” logic gate CAR. **(D)** Derivation of nanobodies for construction of nanoCARs. **(E)** T cell receptor fusion constructs (TRuCs) that contain an ScFv linked to various subunits of an endogenous TCR.

Third-generation CARs utilize a combination of multiple costimulatory signaling domains within the endodomain (**Figure 3**). Some of the common constructs carry CD3ζ-CD28-OX40 or CD3ζ-CD28-4-1BB (14, 24). Amongst these third generation CAR constructs, CD3ζ-CD28-4-1BB seems to be the most promising given the CD28 costimulatory domains contribute to rapid tumor elimination while the 4-1BB endodomains increase CAR survival (25, 26). Third generation CARs have been successful in the treatment of cancers as they demonstrate high proliferation, maintain longer survival periods, and display good safety profiles. However, their efficiency in tumor elimination does not always remain superior compared to second-generation CAR T cells (20). Hence, efforts to improve efficiency and safety resulted in the development of fourth generation CARs.

### 3.4 Fourth generation CARs

Subtle modifications of third generation CAR Ts gave rise to fourth-generation CARs. These CARs feature expression of transgenic proteins, often cytokines, that can be inducibly or constitutively expressed. In some cases, these CAR Ts are also designed with a regulatory switch for a suicide gene or an element that enhances T cell function (27, 28). In one instance, inclusion of Caspase 9 enhanced the safety of CAR Ts (29). An advanced version of this CAR was developed by Labanieh et al. (30). The group designed protease-based CARs that could be regulated with drug doses to enhance the safety and efficacy of the CAR Ts. Also belonging to the fourth generation of CAR Ts are T cells redirected for universal cytokine-mediated killing (TRUCKs). These CAR Ts are engineered to carry a nuclear factor of the activated T cell (NFAT)-responsive cassette (containing a transgenic cytokine such as IL-12) and deliver the transgenic product to the tumor site (**Figure 2**). The presence of a cytokine transgene enhances the efficacy of CAR T cell therapies in preclinical models with low systemic toxicity (31, 32). Thus, in TRUCKs, the expression of the transgene is induced when CD3ζ-containing CARs encounter their specific target. TRUCK CAR T cells thus carry two transgenic cassettes – one for the CAR structure and another for the inducible cytokine.

### 3.5 Fifth generation CARs

Fifth generation CARs integrate an additional membrane receptor into their structure. Of the several different approaches tested, one of the most promising is the addition of IL-2 receptors (**Figure 2**) that allow for antigen-dependent JAK/STAT pathway activation (33).

Another promising approach is the inclusion of switch receptors. Incorporation of a drug-dependent OFF-switch that leads to CAR depletion or the incorporation of an ON-switch that leads to activation remains a promising option. In fact, lenalidomide-gated CARs are designed on this principle (34). Even though these CARs exhibit less efficiency *in vitro*, they remain favored from a research perspective given their increased regulation and enhanced safety profile. Li et al. (2022) further developed this concept by developing a multiantigen targeted collection of CARs controllable with FDA-approved antiviral drugs (35). The VIPER (VersatIle ProtEase Regulatable) CARs are inducible with ON and OFF switch CAR circuits engineered using a viral protease domain. The group demonstrated limited toxicity of their system in a cytokine storm animal model. In addition, they also developed several complex CAR circuits with logic and multiplex control functions.

## 4 Modifications of CAR T cells

### 4.1 Dual-signaling CARs

Dual-signaling CARs make use of two separate CARs on the same T cell with each CAR cell having its own intracellular domains (**Figure 3A**). Such CARs are much more effective since they can target different antigens at the same time. Dual targeting, split costimulatory signaling and shared CD3ζ chain tailored CARs have been used to target two clinically relevant antigens (15). These dual-signaling CARs were targeted against GD2 and B7-H3 in a neuroblastoma disease model (36–38). In fact, this approach was further validated with other pairs of targets, namely mesothelin (MSLN) and chondroitin sulfate proteoglycan 4 (CSPG4) (15).

Similarly, a dual-signaling CAR T targeting CD19 and CD123 was created and proved more efficient in tumor eradication in preclinical trials than using CAR Ts with a single signaling moiety (CD19-CAR T cells and CD123-CAR T cells) (18). Additionally, this dual-signaling CAR T prevented disease recurrence caused by downregulation or loss of target antigens on the tumor cell surface much more efficiently. Thus, it has been shown that the dual-signaling CAR strategy allows for rapid and sustained antitumor effects while preventing tumor escape in heterogeneous antigen expression tumor cell populations (15).

### 4.2 Tandem CARs with multiple single-chain variable fragments

Refinement of antigen binding domains in the form of scFvs has also led to the production of CARs that target more than one antigen in the form of multiple antigen-binding CARs (**Figure 3B**). These CARs exist in the form of dual CARs, consistent of two separate binding domains, and in the form of TandemCARs, which contain a modified CAR ectodomain that encodes for multiple antigen-binding domains in a CAR module using scFvs from monoclonal antibodies (39–41).

To further enhance the efficiency of CARs, antigen binding domains have been designed to carry three scFvs in tandem. As a result, these CARs can target three different tumor associated antigens (TAAs), allowing for their utility in treating tumors with heterogeneous cell population (42). Constructed CARs with a single universal tricistronic transgene were designed to recognize three different TAAs, namely, human epidermal growth factor receptor 2 (HER2), interleukin-13 receptor subunit alpha-2 (IL13Rα2), and ephrin-A2 (EphA2) to treat glioblastoma. The outcomes of this CAR T therapy revealed that the trivalent-tandem CAR had strong anti-tumor activity and could overcome tumor antigen heterogeneity at a level greater than nonspecific or bispecific CAR T cells. On similar lines, Balakrishnan et al. (2019) designed a trivalent-tandem CAR by using three scFvs in tandem with ankyrin repeat proteins (DARPins) (43). These CAR T cells displayed potent anti-tumor effects and performed well despite extensive tumor antigen heterogeneity and immune escape.

### 4.3 CARs with combinatorial antigen sensing circuits

Another modification of CAR involves a design concept in which the CAR T can only be activated when two scFvs bind to corresponding antigens simultaneously (**Figure 3C**). Boolean logic gates are the recent developments to CAR T cells as safety switches. Integrated signals from multiple receptors at once can regulate CAR T cells activity based on their environment. The “AND”- gate logic utilizes two receptors that recognize different tumor antigens. Binding to both antigens of interest is needed to trigger CAR T cell activation (44). This combinatorial antigen sensing circuit is dependent on the synNotch receptor that forms the core design of “AND”-gate CARs. The synNotch binds to the first selective tumor antigen, inducing the expression of the second CAR, which then binds to specific second tumor antigen leading to the activation of CAR T.

The utilization of two scFvs binding to corresponding antigens can also be used to limit on-target off-tumor toxicity. Inhibitory CARs (iCARs) contain a T cell inhibitory signaling domain. An AND-NOT gate CAR T strategy allows for the CAR on the T cell to recognize the tumor antigen and activate the T cell while the iCAR recognizes a normal tissue antigen and inhibits T cell activity (45). This allows the CAR T cell to limit its on-target off-tumor toxicity by distinguishing between a tumor cell and a normal cell that both have the same CAR target antigen.

### 4.4 CARs with variable heavy domains of heavy chains antigen binding domains

While CARs exhibiting a TCR-like antibody form utilize scFvs from monoclonal antibodies, there exist other mechanisms for antigen binding (46). Another approach for CAR binding relies on variable heavy domains of heavy chains (VHHs), solely the variable domains of heavy-chain-only antibodies (HCAbs) (47). Given advancements in the complementarity-determining regions (CDRs) of VHHs, these VHHs, or nanobodies, can bind antigens with affinities comparable to those of antibodies (**Figure 3D**). These binding affinities are enhanced by further mutations in the VHH framework which allow for these molecules to remain stable in aqueous solutions while containing soluble protein domains (48). The applicability of these nanobody-driven CARs, or nanoCARs, is derived from investigations showing the drawbacks associated with scFv framework regions in CARs - such regions have shown to cause CAR aggregation and tonic signaling with low cell activation (49). Although nanoCARs are still subject to this mentioned aggregation issue, nanoCARs remain more predictable in their binding ability given their monomeric structure, allowing for the use of immune libraries (50).

### 4.5 T cell receptor fusion constructs

T cell receptor fusion constructs (TRuCs) utilize the biology of TCRs and fuse them with an antibody-based binding domain (44). The fusion of the antibody-based binding domain to the TCR to create TRuCs allows these modified TCR complexes to effectively recognize surface antigens (**Figure 3E**). These modified TCR complexes, specifically referred to as TRuC-T cells, have shown to kill tumor cells with the same level of potency as second-generation CAR-T cells while displaying a significantly lower cytokine release (51). Additionally, TRuCs have been used in targeting treatment-refractory mesothelin expressing solid tumors through the fusion of an anti-mesothelin antibody to the endogenous TCR complex. This TRuC-T therapy, gavocabtagene autoleucel, has shown positive results in a Phase I/II clinical trial (NCT03907852) has shown promising results with a disease control rate of 77% in thirty patients (52).

## 5 Manufacturing the CAR T

### 5.1 Acquisition of T cells

After leukapheresis separates leukocytes from the blood of patients, enrichment of the product allows for collection of T cells (53). Rather than completing the intensive process of purification of autologous antigen-presenting cells (APCs) from patients to activate T cells, beads coated with anti-CD3 and anti-CD28 monoclonal bodies have been employed to activate T cells in an efficient way (54, 55). Traditionally, autologous T cells, cells purified from the patient to be treated, have been used to produce CARs (56). However, a considerable drawback associated with the usage of autologous T cells remains the retrieval of patient specific T cells for CAR development for effective CAR T therapy. In specific, drawbacks include the time-consumption and cost associated with autologous T cell production, the functional quality of autologous T cells, and the potential manufacturing failures associated with autologous T cell preparation (57, 58). Unlike autologous T cells, allogeneic T cells serve as universal T cells from healthy donors.

**Figure 4:**
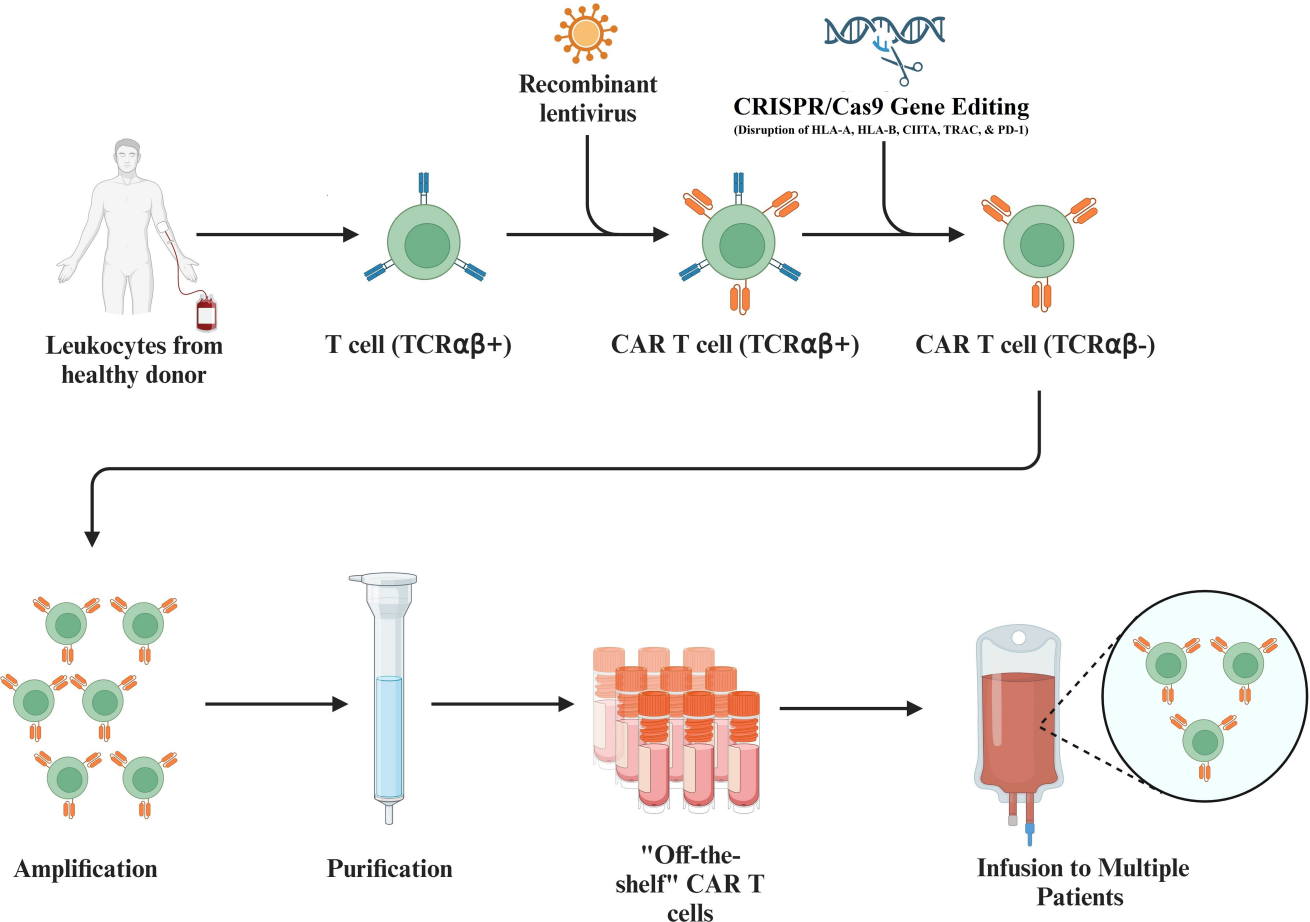
Utilization of Allogeneic T Cells to Produce CAR T Cells. Isolated T cells collected from healthy donor leukocytes allow engineering of the T cells so that they can be used universally. This process includes the introduction of a recombinant lentivirus to add the desired CAR to the T cell and includes the CRISPR/Cas9 mediated disruption of the HLA-A, HLA-B, class II major histocompatibility complex transactivator (CIITA), T-cell receptor alpha constant (TRAC), and Programmed Cell Death Protein 1 (PD-1) on transduced T cells. Further amplification and purification of these allogeneic T cells produces universal CAR T cells that can be infused to multiple patients.

To circumvent graft-versus-host diseases, allogeneic T cells are produced through genetic disruption of HLA-A, HLA-B, class II major histocompatibility complex transactivator (CIITA), T-cell receptor alpha constant (TRAC), and Programmed Cell Death Protein 1 (PD-1), leading to their inability in recognizing allogeneic antigens (57, 59, 60). Allogeneic T cells can thus be used to produce nearly universal CAR T cells for antigens of interest (**Figure 4**). Though these genetic disruptions to the HLA loci, CIITA, TRAC, and PD-1 present on T cells serve to mitigate the effects of graft-versus-host disease, there remains a life-threatening risk of graft-versus-host disease. In addition, universal allogeneic generated CAR T cells are eliminated by the patient’s immune system, whereas autologous CAR T cells last on the timescale of months to years (4, 58).

### 5.2 T cell engineering

Within activation, T cells are subject to incubation with a produced lentiviral vector encoding for the CAR. Viral machinery can be utilized to inject RNA within patient cells such that the RNA is reverse transcribed into patient T cell DNA. This reverse transcription encodes for the CAR and allows for maintained expression, via transcription and translation, of the CAR within the patient T cells as they are expanded (61, 62). While lentiviral vectors are commonly employed to express CARs on patient T cells, manufacturing of the CAR T cell can also be completed by utilizing mRNA transfection and the Sleeping Beauty transposon system.

mRNA based transfection utilizes electroporation to modify T cells with transient CAR expression (63). This approach yields significant antitumor effects in preclinical models of solid tumors like mesothelioma, while remaining a cost-efficient manner to manufacture CAR T cells (64). One drawback, however, is that the mRNA transfection approach requires multiple rounds of CAR T cell infusion (65). Additionally, *in vivo* delivery of mRNA can be mediated by lipid nanoparticles in a non-viral vector system (66). These lipid nanoparticles-mRNA formulations protect the mRNA from nuclease degradation in cellular fluids while avoiding clearance by renal glomerular filtration as mRNA is internalized by target cells (67). Lipid nanoparticles have been administered in the clinical setting for small molecule and mRNA delivery (68).

The Sleeping Beauty transposon-based system utilizes electro-transfer of non-viral plasmids for DNA transduction - this clinical grade production occurs at a cost that is nearly 10% of the cost associated to produced good manufacturing grade (GMP) virus. Additionally, with proven potential for human gene therapy, the Sleeping Beauty system can generate significant amounts of modified T cells within 28 days (69, 70). Further positives associated with the Sleeping Beauty transposon system include its relative inexpensiveness in early-stage clinical trials. However, there remain lingering questions regarding the efficacy of this system in comparison to lentiviral vectors and the potential remobilization of transposons (71).

### 5.3 *In vivo* manufacturing

CAR T cells can be produced *in vivo* using various techniques. The first of these techniques employs the utilization of lentiviral vectors. Though the lentiviral vector approach can be used to manufacture CAR T cells *in vivo*, the tropism of CAR-encoding lentiviral vectors to T cells must be confined. This can be done by replacing natural receptor usage of the vectors and fusing binders which recognize solely T lymphocyte binders, such as CD8 or CD4 (72–74). Lentiviral vectors have been used to generate human anti-CD-19 CAR T cells by targeting human CD8+ cells in a mouse model (75). While such an approach did lead to B-cell depletion and signs of cytokine release syndrome (CRS), the utilization of CD4 lentiviral vectors led to the production of anti-CD-19 CD4+ CAR T cells that led to more efficient tumor cell killing activity (76).

Adeno-associated virus (AAV) vectors can also be used to manufacture CAR T cells *in vivo*. The injection of an AAV vector containing the CD4 targeting CAR gene allowing for the generation of potent CAR T cells *in vivo* that ultimately led to tumor regression in a mouse model (77).

Non-viral approaches have also been established in the *in vivo* manufacturing of CAR T cells. Lipid nanoparticles (LNPs) can be used to transfect T cells with mRNA *in vivo* through conjugation to antibodies or ligands that target various receptors on these T cells, including CD3, CD8, CD4, CD7, and CD5 (78–83). Specifically, LNPs using antibody conjugation platforms have been used to selectively and efficiently produce functional CAR T cells *in vivo* through the use of CD3 and CD7-LNPs (84).

### 5.4 *Ex vivo* manufacturing

While the methods to manufacture the engineered CAR T cell therapy are further expanded and refined, there remains the manufacturing bottleneck of growth of large enough numbers of cells for clinical use with the use of bioreactor culture systems (85). There are significant costs associated with both the aforementioned leukapheresis and expansion steps. While methods and devices, like the G-Rex (Wilson-Wolf) exist to expand cells from low seeding densities, propagation of such devices pose an obstacle to the widespread manufacturing of CAR T cells (86). However, devices like the CliniMACS Prodigy, produced by Miltenyi Biotec, provide hope for the future given its ability to prepare, enrich, activate, transduce, expand, and sample cells (87). While expensive, this device has been used to produce autologous CD19 CAR T cells safely and efficiently for treatment of lymphomas, making it plausible for use in clinical trials (88, 89).

Rapid manufacturing of CAR T cells also remains a prominent area of research and development. The typical ex vivo manufacturing of T cells involves activation, viral transduction, and ex vivo expansion for at least six days – this time in activation and expansion can negatively affect CAR T cell therapies due to the progressive differentiation and loss of anti-leukemic activity of these CAR T cells over that time (90). Utilizing lentiviral vectors to transduce non activated quiescent T cells allows for the stable expression of a CAR in these non-activated T cells. This process saves time and resources while also promoting higher efficacy at lower doses as compared to those seen in activated T cells (90, 91).

## 6 *In vivo* CAR T studies

Tumor heterogeneity is an evolving process that hinders tumor eradication by various therapeutic approaches. Using multiple CAR T cells targeting a specific single antigen is one of the options to overcome the problem of tumor heterogeneity. Co-administration of PSCA and MUC1 targeted CAR T cells showed a tumor-killing effect in an *in vivo* mice model implanted with non-small cell lung cancer (NSCLC) cells (92). At the same time, cytokines in the immunosuppressive tumor microenvironment render CAR T cells non-functional. Reversing the suppressive cytokine pathways is a strategy to overcome these challenges. Engineered TGF-β dominant-negative receptors (DNR) display inhibitory action on TGF-β signal (93). This action blocks the transformation of primitive T cells into Treg. TGF-β receptor II (TGFBR2) expressing CAR T cells engineered with CRISPR/Cas9 technology showed anti-tumor activity in *in vivo* models (94). Mesothelin targeting CAR T cells with PD-1 dominant negative receptor (DNR) expression had increased activity in pleural mesothelioma xenograft models (95). FAS DNR expressing CAR Ts showed a similar result in an *in vivo* model (96).

In another study, CAR T cells with inducible IL-12 secretion upon target encounter were developed to overcome the systemic toxicity of constitutively active IL-12 (31, 32). In these studies, the induction of IL-12 in CAR Ts reduced antigen-positive tumor growth and prevented the growth of antigen-free cancer cell proliferation in mice compared to CAR T cells lacking the cytokine. There was also increased macrophage accumulation at the tumor site in the presence of IL-12 thereby promoting anti-tumor activity (31). Additionally, the engineering of an optimized membrane-bound IL-12 molecule in CAR T cells led to positive results in *in vitro* and *in vivo* models. Specifically, CAR T cells equipped with the membrane bound IL-12 molecule showed increased antigen-dependent T cell proliferation and showed high efficacy in human ovarian cancer xenograft models. Membrane-bound IL-12 also promoted durable anti-tumor responses with demonstrated safety and efficacy in an immunocompetent mouse model (97).

Administration of anti-CD19 CAR T cells with constitutive expression of IL-18 to mice bearing CD19+ tumors showed better survival of mice compared to CAR Ts without this feature (98). The survival was attributed to CAR Ts increased cell expansion and persistence and activating the endogenous immune system. As opposed to constitutive expression, inducible IL-18 CAR T cells also showed reduction in the size of advanced pancreatic tumors in mice and prolonged survival compared to treatment with the CAR alone (99). It was also observed that IL-18 producing CAR T cells were more effective than IL-12 producing CAR Ts in controlling late-stage cancer. Similarly, anti-Delta-like Protein 3 (DLL3) IL-18 secreting CAR Ts were shown to have an enhanced potency, as well as increased activation of CAR T cells and tumor-infiltrating lymphocytes, in small cell lung cancer models (100).

CAR T cells engineered to secrete IL-15 have been shown to exhibit increased tumor cytotoxicity compared to using CAR alone T cells *in vitro* and *in vivo* studies (101, 102). The secretion of IL-15 provided greater protection against tumor rechallenge. It was also observed that IL-15 tethered to the membrane promoted the CAR T cells to develop a memory phenotype.

## 7 Current antigens and the search for new antigens

The first U.S Food and Drug Administration (FDA) approved CAR T cell therapy targets the B-lymphocyte antigen CD19, a transmembrane glycoprotein that belongs to the immunoglobulin superfamily. CD19 functions as a biomarker for follicular and dendritic cells and for neoplastic and normal B cells (92). Working as part of an extracellular complex on the surface of mature B cells, CD19 functions as an integral signaling component of these cells (103). Given the universal expression of CD19 in B cell neoplasms, CD19 has been the first target of CAR T cell development. Since the approval of CD19-targeted CAR T cell therapy for patients with acute lymphoblastic leukemias (ALL), the indication has expanded to aggressive B cell lymphomas. The current approved CD-19 targeted CAR T cells include axicabtagene ciloleucel, brexucabtagene autoleucel, tisagenleucelucel, and lisocabtagene maraleucel (4, 6, 7, 104–110). In fact, the effectiveness of CAR T cells targeting CD19 in ALL has been exceptional (111). Following the approval of CAR T cell therapies targeting CD19, the FDA has since approved CAR T cell therapies targeting B-cell maturation antigen (BCMA) for patients with multiple myeloma (MM) (112). MM is a malignant disease defined by the proliferation of plasma cells in bone marrow (113). With the continuous increase in the prevalence and frequency of MM, BCMA has become an antigen of significant interest, given its expression on the surface of both B-cell lineage cells and malignant plasma cells (114, 115). Given BCMA’s role in cell survival and its expression on the surface of target cells, it remains a pivotal antigen for the development of CAR T cell therapies (116, 117). Studies have demonstrated the significant clinical activities of BCMA-targeted CAR T cells, leading to the approval of idecabtagene vicleucel (ide-cel) and ciltacabtagene autoleucel (cita-cel) (8, 118).

Given the potential robustness of CAR T cell therapy, there remains a search for additional tumor specific antigens. Of paramount importance in utilizing CAR based therapy for solid tumors is the distinction of antigens that would allow the CAR to be specific and effective in recognizing tumorous cells (119). The difficulty that arises in identifying such antigens is due to potential off-tumor on-target toxicity.

T cell receptor-engineered T (TCR-T) cell therapy is another rapidly developing form of adoptive cell therapy that targets tumor antigens. Unlike CAR therapy, TCR therapies rely on the MHC mediated presentation of peptide sequences from antigens (120). Such an approach can be advantageous as TCR-T therapy is not limited solely to antigens presented on the surface of cancer cells (121). However, there have been documented risks associated with the potential adverse effects of TCR and tumor associated antigen (TAA) mispairings. Additionally, there remain drawbacks given the difficulty associated with TCR acquisition (122). An approach where a TCR-like antibody is fused with CD28 and CD3-zeta chain endo-domains allows for the expression of TCR-like CARs on the surface of T cells. With TCR-like antibodies fused with transduction signals, these TCR-like CARs are then able to mediate interactions between the engineered T cells and tumor cells (123). Such an approach allows for TCR-like antibodies against peptide sequences presented on the ectodomains of tumor cells. This allows the TCR-like CAR to bind to directed antigens independent of the MHC-I, allowing for the activation of the intracellular domain signal transduction system which leads to the lysis of target tumor cells (123). The direct binding of a CAR to its antigen, a process that is mediated by TCR-like antibodies, allows for the engineered T cell to receive a fully competent activation signal - such a signal allows for the ensuing CAR dependent killing and cytokine production (69).

The exploration of CARs that bind to antigens based on natural affinity is also underway (124). Such CARs, natural ligand-receptor CARs, utilize molecules that maintain a natural affinity for an associated target. Unlike previously mentioned CARs, this binder does not rely on foreign antibody introduction. First generations of CARs targeting stress-associated ligands from NK cell receptors exhibit universal behavior across tumor types (125, 126). Though a certain drawback of ligand-receptor CARs is the lack of tumor restrictive ligand expression, there remains increased risks of on-target off-tumor toxicity, as shown with CARs targeting CD70 using the costimulatory receptor CD27 (127, 128). However, a distinct advantage of these CARs remains their lowered immunogenicity given the lack of refinement of extracellular components (129).

Given this overview of antigen binding, identification of tumor specific antigens remains one of the biggest challenges in the development of CAR therapy. Pan-cancer analyses have shown that tumor tissues contain up to 30% more alternative splicing events as compared to normal tissues (130). Findings like these have garnered interest in characterizing alternative splicing events as potential targets for immunotherapy (111, 112). A recently developed method to identify new antigens for CAR therapy comes in the form of splice isoforms found on the ectodomain of cells (16). The development of a computational platform, Isoform peptides from RNA splicing for Immunotherapy target Screening (IRIS), has potential in allowing for the discovery of tumor specific antigens. Long read RNA sequencing allows for the discovery of mRNA splice junctions. Such tumor-associated splice-junctions are then screened against pre-compiled tumor and normal transcriptomes. The approach to determine splice isoforms on cellular ectodomains in solid tumors based on sequencing data of selected cancer lines has allowed for an expansion in the realm of potential antigens for CAR therapy (131, 132). Additionally, this expanded range of potential antigens is coupled with the discovery of potentially novel splice isoforms of protein that are unique to specific solid tumors (16). In turn, these splice isoforms on cellular ectodomains could serve as an expanded range of potential antigens that also lower the risk of on-target off-tumor toxicity given their lack of expression in normal tissues (133).

## 8 Antigen targets in CAR T clinical trials

There are numerous CAR T clinical trials targeting various antigens for solid tumor treatment. These antigens, along with information regarding current and past clinical trials targeting these antigens in solid tumors, are examined in this section and are represented in **Table 1**.

**Table 1:** The list of active or completed phase I/II clinical trials of CAR T cell therapies by target antigen.

Target antigen	Trial no.	Disease	CAR T therapy cell source	Co-targeted antigens	Phase	Recruitment status
EGFR	NCT01869166	EGFR positive advanced solid tumors	Autologous		Phase 1 & 2	
	NCT03182816	EGFR positive advanced solid tumors	Autologous	CTLA-4 and PD-1	Phase 1 & 2	
	NCT03941626	Esophageal, hepatoma, glioma and gastric cancer	Autologous	NY-ESO1, DR5, and mesothelin	Phase 1 & 2	
Her2	NCT04650451	Advanced Her2 positive solid tumors	Autologous		Phase 1	Discontinued due to DLT
	NCT01935843	Advanced Her2 positive solid tumors	Autologous		Phase 1/2	
	NCT04511871	Advanced Her2 positive solid tumors	Autologous		Phase 1	Active, recruiting
CEA	NCT06006390	CEA positive advanced solid tumors			Phase 1/2	Recruiting
	NCT06043466	CEA positive advanced solid tumors			Phase 1	Recruiting
	NCT05538195	CEA-positive advanced solid tumors			Phase 1/2	Recruiting
	NCT05736731	Solid tumors that express CEA and have lost HLA-A*02 expression	Autologous	Absence of HLA-A*02	Phase 1/2	Recruiting
	NCT06126406	CEA positive advanced solid tumors			Phase 1	Recruiting
Mesothelin	NCT03054298 and NCT03323944	Advanced solid tumors expressing mesothelin	Autologous		Phase 1	Active, not recruiting
	NCT03373097	High risk and/or relapsed/refractory neuroblastoma	Autologous		Phase 1 & 2	Recruiting
	NCT03182803	Mesothelin positive advanced solid tumors	Autologous	CTLA-4 & PD-1	Phase 1/2	
	NCT03941626‡	Advanced refractory solid tumors	Autologous	NY-ESO1, DR5, and mesothelin	Phase 1/2	
	NCT03545815	Advanced mesothelin positive solid tumors	Autologous		Phase 1	
	NCT03030001	Advanced mesothelin positive solid tumors	Autologous		Phase 1/2	
	NCT03356795‡	Cervical cancer		GD2, PSMA, Muc1	Phase 1/2	
ROR1	NCT05274451	Advanced lung cancer and TNBC	Autologous		Phase 1	Recruiting
	NCT02706392	Advanced ROR 1 positive solid tumors	Autologous		Phase 1	Terminated due to slow accrual
	NCT05694364	Advanced hematologic and solid malignancies	Autologous		Phase 1	Recruiting
ROR2	NCT03960060	Recurrent or refractory solid tumors	Autologous		Phase 1	
Claudin 18.2	NCT03874897	Advanced solid tumors; gastric cancer in phase 2	Autologous		Phase 1	Recruiting
	NCT05393986	Advanced solid tumors	Autologous		Phase 1	Recruiting
	NCT05472857	Advanced CLDN 18.2 positive gastric cancer	Autologous		Phase 1	Recruiting
	NCT05952375	Advanced CLDN 18.2 positive solid tumor			Phase 1	Recruiting
GD2	NCT03373097‡	High risk and/or relapsed/refractory neuroblastoma or other GD2-positive solid tumors	Autologous		Phase 1/2	Recruiting
	NCT04196413	Diffuse intrinsic pontine gliomas with H3K27M mt and spinal diffuse midline glioma with K27M mt	Autologous		Phase 1	Recruiting
	NCT02107963	Advanced GD2 positive solid tumors	Autologous		Phase 1	Completed
	NCT01822652	Relapsed or refractory neuroblastoma	Autologous		Phase 1	Active, not recruiting
	NCT03635632	Relapsed or refractory neuroblastoma and other GD2 positive cancers	Autologous		Phase 1	Active, not recruiting
	NCT02992210	GD2 positive solid tumors	Autologous		Phase 1/2	
	NCT03356795‡	Cervical cancer		Mesothelin, PSMA, Muc1	Phase 1 & 2	
MUC1	NCT04025216	TnMUC1-positive advanced cancers	Autologous		Phase 1	Terminated due to unfavorable result
	NCT03179007	MUC1 positive advanced solid tumors	Autologous	Co expressing CTLA-1 and PD-1	Phase 1 & 2	
	NCT03356795‡	Cervical caner		GD2, mesothelin, and PSMA	Phase 1 & 2	
	NCT02617134	MUC1 positive solid tumors	Autologous		Phase 1 & 2	
B7H3	NCT05190185	B7-H3 positive advanced solid tumors			Phase 1	
	NCT05515185	B7-H3 positive solid tumors	Autologous		Phase 1	Not yet recruiting
	NCT04864821	B7H3 positive advanced solid tumors	Autologous		Phase 1	
	NCT04897321	B7H3 positive solid tumors in pediatric	Autologous		Phase 1	Recruiting
	NCT04691713	Advanced CD276+ solid tumors	Autologous		Phase 1	Recruiting
GPC3	NCT02932956	GPC3 positive pediatric solid tumors	Autologous		Phase 1	Active, not recruiting
	NCT04715191	GPC3 positive pediatric solid tumors	Autologous		Phase 1	Not yet recruiting
	NCT04377932	GPC3 positive pediatric solid tumors	Autologous		Phase 1	Recruiting
	NCT05103631	GPC3-positive solid tumors	Autologous		Phase 1	Recruiting
GCC	NCT05287165	GCC positive advanced digestive system neoplasms			Phase 1	Recruiting
	NCT05875402	GCC positive recurrent or refractory solid tumors			N/A	Recruiting
PSCA	NCT02744287 and NCT04650451	Metastatic castration resistant prostate cancer	Autologous		Phase 1/2	Suspended due to DLT
	NCT03873805	Metastatic castration resistant prostate cancer	Autologous		Phase I	Completed
PSMA	NCT04249947	Metastatic castration-resistant prostate cancer and advanced salivary gland cancers	Autologous		Phase 1	Active, not yet recruiting
	NCT03089203	Metastatic castration-resistant prostate cancer	Autologous	TGFβ-resistant	Phase 1	Active, not yet recruiting
NKG2D	NCT04107142	Relapsed or refractory solid tumors	Haplo/Allogeneic		Phase 1	
	NCT05302037	Advanced solid tumors	Allogeneic		Phase 1	Not yet recruiting
EphA2	NCT05631886	Advanced solid tumors	Autologous		Phase 1	Recruiting
	NCT05631899	Advanced solid tumors	Autologous		Phase 1	Recruiting
EpCAM	NCT02915445	Advanced solid tumors	Autologous		Phase 1	Active, not yet recruiting
CD22	NCT04556669	Advanced solid tumors	Autologous		Phase 1	Recruiting
CD70	NCT02830724	CD70 expressing cancers	Autologous		Phase 1 & 2	Recruiting
Lewis Y	NCT03851146	Lewis Y expressing solid tumors	Autologous		Phase 1	Completed

‡ These trials have multiple targets.

### 8.1 EGFR

The Epidermal Growth Factor Receptor (EGFR) is considered an important target for anti-tumor therapy in many solid tumors such as non-small cell lung cancer, gastrointestinal cancer, and colorectal cancer. Ongoing clinical studies are evaluating CAR T cell therapy targeting EGFR. In the case of NSCLC, a phase I trial for patients with EGFR mutation (NCT01869166) enrolled fourteen patients. Four of fourteen patients showed partial response for two to four months, and eight patients showed stable disease for two to four months as well. All Grade 3 events were manageable. The progression-free survival (PFS) was three months, and the overall survival (OS) was 4.9 months (134). Another phase I trial for patients with refractory/relapsed NSCLC showed therapeutic response (NCT03182816). One of nine patients maintained partial response for more than thirteen months, six patients showed stable disease, otherwise, and two patients showed progression in disease. All Grade 1-3 adverse events were manageable with proper treatment. The PFS was 7.13 months and OS was 15.63 months (135). An open label phase I clinical trial for patients with glioblastomas with EGFRvlll mutation (NCT02209376) assessed the efficacy of autologous CAR T cells targeting EGFRvlll, however, it was terminated prior to completion due to futility (136).

### 8.2 HER2

Human Epithelial Growth Factor Receptor 2 (HER2) plays a key role in targeted therapy, especially in breast cancer, and gastrointestinal malignancies. In addition, other types of solid tumors are applicable to Her2 targeted therapy and researchers highlighted the potential of HER2-targeted-CAR T cell therapy. A phase I trial of HER2-specific dual-switch CAR T cells in previously treated HER2 positive solid tumors (NCT04650451) showed the therapeutic and safety profile of CAR T cell products in combination with rimiducid. Five of nine patients achieved a prostate specific antigen (PSA) 50 response, and four of nine patients also got a PSA90 response. However, the last patients with metastatic castration-resistant prostate cancer experienced Grade 4 cytokine release syndrome (CRS) and reached second dose limiting toxicities (DLT) in the dose-escalation cohort. Given this safety issue, the trial was terminated. A phase I trial (NCT0195843) revealed that HER2 directed CAR T had the activity in advanced biliary tract cancer and pancreatic cancer. One patient achieved partial response for 4.5 months and five obtained stable diseases. Regarding safety profiles, most adverse events (AEs) were Grade 1 or 2, except one Grade 3 febrile syndrome and one abnormal liver function test (LFT) elevation that were both manageable (137). An ongoing phase I trial (NCT04511871) was designed to evaluate the efficacy of CCT 303-406, a HER2 targeting CAR T in refractory HER2 positive tumors. The primary objective was safety, tolerability, and optimization of the dose for phase II trial. In 2023, a new phase I trial (NCT05745454) began to recruit patients with HER2 positive solid tumors.

### 8.3 CEA

Carcinoembryonic Antigen (CEA) is an established tumor marker in epithelial solid tumors including colorectal cancer (CRC). As a result, CEA is regarded as a promising target for CAR T therapy. A phase I/II trial, which began to recruit patients in August 2023. is underway to assess safety and tolerability of CEA-targeted CAR therapy for patients with CEA-positive advanced solid tumors such as gastrointestinal (GI) malignancy, lung cancer, and breast cancer (NCT06006390). Another ongoing phase I trial (NCT06043466) is enrolling patients with various solid tumors, including GI malignancies and NSCLC. The primary objectives are determining the DLT and the maximum tolerated dose (MTD), while secondary objectives include disease control rate (DCR) within three months, maximum concentration of dosage, maximum time to reach the highest concentration, and the content of CEA in peripheral blood after infusion of the CAR T cells. Multiple other trials are evaluating the safety and efficacy of CEA-targeted CAR T cell therapy for patients with CEA- positive advanced solid malignancies (NCT05538195, NCT06126406). Lastly, a logic-gated Tmod CAR T therapy of A2B530 showed its safety and efficacy in patients with solid tumors associated with CEA expression and loss of heterozygosity (LOH) of HLA-A*02 (NCT05736731, EVEREST-1). The primary objectives of this phase I study are to assess the safety and tolerability of this therapy in patients with NSLC, CRC, and pancreatic cancer, while determining the maximum tolerated dose and the recommended phase II dose (RP2D).

### 8.4 Mesothelin

Mesothelin (MSLN) is usually expressed in normal mesothelial cells; however, it is overexpressed in pancreatic cancers, ovarian cancers, and mesothelioma as well. MSLN is considered as a potential target for CAR T cell therapy and multiple trials tested its clinical activity (138). A phase I of MSLN-targeting CAR T cells demonstrated anti-tumor activity in pancreatic ductal carcinoma, ovarian cancer, and malignant mesothelioma (NCT03054298 and NCT03323944). In these studies, eleven of fifteen patients obtained stable disease (the best response), and one patient experienced dose-limiting toxicity of Grade 4 sepsis. Short persistence after the intravenous infusion of the CAR T cells appears to be associated with limited clinical activity. Ongoing studies are evaluating the regional delivery (intrapleural administration, intramural administration, etc.) of CAR T cells to enhance their proliferation, persistence, and function (139). MSLN-targeting CAR T cells (mesocarp-T) that express CTLA-1 and PD-1 antibodies are under evaluation for patients with various MSLN positive solid malignancies (NCT03182803). Other early phase trials (NCT03941626) aimed to evaluate MSLN-targeted CAR T therapy in hepatocellular carcinomas, glioblastomas, esophageal carcinomas, and gastric carcinomas, but their results remained veiled so far (140–142).

### 8.5 ROR1

Receptor tyrosine kinase-like orphan receptor (ROR) is a subfamily of RTK and serves as a nuclear receptor interacting with intracellular transcription factor. It plays a significant role in embryonic development, and numerous studies have shown its aberrant expression in many diseases including advanced lung and triple negative breast cancers, making it a candidate for CAR T therapy (143, 144). A ROR1 targeted CAR T cell therapy, LYL797, is being tested in patients with advanced lung and triple negative breast cancers (NCT05274451). This phase I trial aimed to assess safety and determine the RP2D. The study plans to enroll fifty-four patients in dose-escalation and expansion cohorts. Another phase I trial of autologous T cell therapy for patients with ROR1+ triple negative breast cancer and ROR1+ non-small cell lung cancer (NCT02706392) assessed the safety profile in six patients, and no DLT was observed. However, the trial was terminated prematurely due to slow accruals (145). A trial of PRGN-3007 UltraCAR-T cells (NCT05694364) targeting ROR1 is currently under clinical development for patients with hematologic malignancies as well as ROR1+ triple negative breast cancers. The UltraCAR-T platform is designed to address various limitations of traditional CAR T cell manufacturing by using a nonviral gene delivery system and an overnight manufacturing process (146). In addition, PRGN-3007 is designed by incorporating Intrinsic down-regulation of PD-1 expression into a CAR T cell. The first patient was enrolled in April 2023 and a total of eighty-eight patients are expected to participate in this study. ROR2 has been considerably less studied than ROR1, however, various studies showed that it is highly expressed in solid tumors including melanoma, osteosarcoma, renal cell carcinoma, and head and neck squamous cell carcinoma (147). Hence, ROR2 has emerged as a candidate for CAR T cell therapy. A phase I trial of CCT301-59 (NCT03960060) is investigating the efficacy and safety of autologous ROR2-targeting CAR T cell therapy in adults with relapsed and refractory stage IV ROR2 metastatic solid tumors. The results have not yet been reported.

### 8.6 Claudin 18.2

Claudin-18.2 (CLDN 18.2) is a member of the claudin family and is expressed in gastric and pancreatic adenocarcinoma (148). Thus, CLDN is a promising target for CAR T cell therapy. A multicenter phase IB trial of salvage CT041 CLDN 18.2 specific CAR T therapy (NCT03874897) against CLDN18.2-pisitve advanced gastrointestinal cancers indicated that the investigational drug had tolerable safety profile; there was a Grade 3 or higher hematologic toxicity and Grade 1/2 CRS in most patients. The overall response rate (ORR) and disease control rate (DCR) were 48.6% and 73.0%, respectively. Patients with gastric cancer showed a better result in ORR and DCR (57.1% and 75.0% respectively). Encouraged by this result, a phase II trial was initiated in heavily pretreated patients with gastric cancer (149). An ongoing autologous T cell therapy of CT048 targets CLDN 18.2 in gastroesophageal (GE) junction cancer, gastric cancer, and pancreatic cancer (NCT05393986). Another phase I trial (NCT05472857) is evaluating the safety and efficacy of IMC002, an autologous CAR T cell therapy, in patients with CLDN 18.2-positive solid tumors. An exploratory trial is currently enrolling patients to assess the safety profile of XKDCT086 (iPD-1-claudin 18.2 CAR T) (NCT05952375) in patients with CLDN 18.2 positive gastric cancer.

### 8.7 GD2

Glycoprotein tumor antigen (GD2) is a membrane protein overexpressed in tumors from neuroectodermal origin (150). Neuroblastoma, retinoblastoma, and melanoma are examples of highly GD2 expressing tumors. Given its limited overall expression in normal tissue and high expression in the aforementioned tumor types, GD2 is considered as a viable candidate for targeted therapy. Since 2018, an ongoing phase I/II trial of autologous GD2-targeting CAR T cells (NCT03373097) is assessing the safety and efficacy of the therapy in pediatric patients with high-risk neuroblastoma (109). Twenty-seven patients received GD2-CART01 and no DLTs were observed. CRS was observed in twenty out of twenty-seven patients, however, nineteen out of twenty experienced a mild form of CRS. ORR was 63% (seventeen of thirty patients); nine patients presented with a complete response (CR), and eight patients presented with partial response (PR). Additionally, a GD2-targeting CAR T therapy (NCT04196413) indicated clinical efficacy in young adults with H3K27M-mutated diffuse intrinsic pontine glioma and spinal diffuse midline glioma. Ten of twelve patients showed improvement in clinical and radiologic courses without severe toxicity. Third generation GD2 CAR T cells were tested in patients with GD2 positive neuroblastoma and osteosarcoma (NCT02107963). Thirteen patients received the drug. These patients maintained stable disease by day 28 and, ultimately, progressed in their disease (151). Conversion between monocyte and myeloid cells may have played a role in limited expansion and activity of the CAR T cells in this trial. Andras et al., conducted a phase I trial to evaluate the clinical benefit of GD2 CAR and iCaspase suicide containing T cells (NCT01822652) in patients with neuroblastomas. The efficacy of C7R-GD2 CART cells has been under investigation in targeting neuroblastoma and other GD+ solid tumors (NCT03635632). A fourth generation GD2 specific CAR T cell therapy (4SCAR-GD2) is also one of investigational drugs for GD2-positive solid tumors (NCT02992210). In the case of cervical cancer, multiple antigens are being examined for patients presenting with GD2, PSMA, Muc1, mesothelin, or other markers (NCT03356795).

### 8.8 MUC1

Glycoprotein Mucin 1 (MUC1) is a member of the mucin family, which consists of mucus for lubricating and protecting normal epithelium. However, MUC1 promotes cancer invasion, metastasis, and neovascularization when it is expressed in cancer cells (152). A study of CAR T-TnMUC1 (NCT04025216) tried to assess the safety, feasibility, and efficacy of autologous T cells in TuMUC1 mutated tumors. Initially, six patients were enrolled, and two patients reached the planned dose. No significant toxicity profile was reported at the initial analysis. The study was terminated in 2023 due to an unfavorable risk-benefit analysis. A phase I/II study of autologous T cells co-expressing immune checkpoint antibodies (CTLA-4 and PD-1) and MUC1-targeting CARs was conducted in patients with MUC1 positive advanced solid tumors (NCT03179007). Another phase I/II trial (NCT02617134) evaluated MUC1-tageting CAR T cells for patients with MUC1 positive solid tumors. The enrollment status of the study is unknown, and the report is not available.

### 8.9 B7-H3 (CD276)

B7-H3(CD276) is a regulatory protein involved in the immune checkpoint pathway. It facilitates immune evasion of cancer cells and leads to proliferation, metastasis, and drug resistance. Given these findings, B7-H3 is a promising target for immunotherapy (153, 154). A TAA06 injection (B7-H3 targeting CAR T cell) trial (NCT05190185) was conducted for patients with malignant melanoma, lung cancer, and colorectal cancer. The enrollment status for this study is unknown. Another B7-H3 targeted autologous CAR T cell trial is planned (KT095 CAR T injection, NCT05515185). For pediatric patients with adrenocortical carcinoma, a trial of B7-H3-targeting autologous CAR T cell therapy is now recruiting patients (NCT04897321).

### 8.10 GPC3

The glypican family is defined by the heparan sulfate proteoglycans consisting of six members (GPC1 to 6). It is related to regulation of Wnt hedgehogs, and fibroblast growth factors and bone morphogenetic proteins signal (155). GPC3-targeting CAR T cells are being tested in children with relapsed or refractor liver tumors (NCT02932956, GAP trial). An Interleukin -15 and -21 armored GPC3 targeted CART therapy will be ready to open (NCT04715191). For other pediatric GPC3-positive solid tumors, interleukin-15 armored GPC3 specific CAR T therapy is recruiting patients. (NCT04377932). An ongoing trial is also assessing T cells engineered with GPC3-CAR and Interleukin-15 (CATCH T cells) in patients with GPC3-positive solid tumors (NCT05103631).

### 8.11 PSCA

Prostate stem cell antigen (PSCA) is a glycosylphosphatidylinositol (GPI) anchored cell surface antigen. It is expressed in epithelial cells of the genitourinary tract organ, skin, esophagus, stomach, and placenta. Overexpression of this antigen is observed in prostate cancer, bladder cancer, renal cell cancer, and hydatidiform mole (156). The results of a phase I trial of BPX 601 in prostate cancer were reported (NCT02744287). A total of eight patients were enrolled, and one patient gained sustained response (SD) after nine months. All patients experienced CRS (6 G1, 2 G2), and one patient reached DLT of neutropenic sepsis (G5). Due to the DLT of neutropenic sepsis, investigators suspended the trial. Additionally, a phase I trial of PSCA-CAR T cells was performed to assess the safety and tolerability (dose limiting toxicity) and determine the RP2D in patients with PSCA positive metastatic castration resistant prostate cancer (NCT03873805). A total of fourteen patients received CAR T cell infusion with varying treatments – the three cohorts were treated with the starting dose level (DL1, 100 million CAR T cells), the starting dose level that included the incorporation of lymphodepletion (DL2), and the starting dose level using a reduced lymphodepletion regimen (156). No DLTs were observed at DL1, while a DLT of grade 3 cystitis was observed at DL2, which led to the addition of DL3 which had no observed DLTs. CRS of grade 1 or 2 observed in five patients; prostate-specific antigen declines occurred in four patients with observed radiographic improvements. While limited persistence of CAR T cells was seen beyond twenty-eight days post-infusion, there were indicative changes as a result of therapy. Such changes included activation of peripheral blood endogenous and CAR T cell subsets, change in the tumor microenvironment, and TCR repertoire diversity in a subset of patients (156).

### 8.12 PSMA

Prostate specific membrane antigen is expressed at high levels in metastatic castration-resistant prostate cancer and has been established as a tumor-associated antigen for immune therapy (157). A phase I clinical trial of autologous anti-PSMA CAR T therapy in metastatic castration-resistant prostate cancer looked to determine the efficacy and safety of the treatment (NCT04249947). In this study, thirteen patients were treated with the P-PSMA-101 autologous CAR-T therapy. Declines in prostate specific antigens were seen in seven patients. CRS was seen in six of patients (one Gr 3 or greater). DLT was seen in one patient with macrophage activation syndrome (Gr 3 or greater CRS event) (158). Additionally, a first-in-human phase I trial of castration-resistant prostate cancer directed CAR T cells, targeting PSMA, and equipped with a dominant negative TGFβ receptor looked to determine the safety, bioactivity, and disease response. A total of eighteen patients were enrolled, and thirteen received varying doses of therapy (NCT03089203). Five of thirteen patients developed greater than grade 2 CRS – one of these patients experienced clonal CAR T cell expansion and death following grade 4 CRS with sepsis (157).

### 8.13 Additional CAR T Antigens

**Guanylyl cyclase 2C (GCC)** is a transmembrane receptor for an enterotoxin produced by diarrheagenic enteric bacteria. The receptor has a unique expression pattern in colorectal cancer (159). Therefore, a GCC-targeting CAR T therapy was tested in patients with metastatic colorectal cancer (NCT05287165). Nine patients received the treatment, and five of nine patients developed GR 1 - 2 cytokine release syndrome. The DCR was 66.7% and ORR was 11.1%. No DLT was observed. These results will open a dose-expansion cohort. In addition, the investigational drug XKDCT080 is recruiting candidates with GCC positive advanced solid tumors (NCT05875402).

**Tyrosinase Related Protein 1 (TYRP1)** is a key protein in melanine synthesis, and it is present in the membranes of normal and malignant melanocytes (160). Therefore, targeting TYRP1 was considered as a potential therapeutic approach in patients with malignant melanoma, and pre-clinical studies were performed to prove its efficacy. A recent report by Ribas and Puig-Saus et al., showed that TYRP1 targeting CAR T cell therapy demonstrated significant antitumor in tumor cells with high TYRP1 expression in murine and patient-derived cutaneous, acral, and uveal melanoma models. The study did not observe significant toxicities and clinical translation is underway based on the efficacy and safety profile (161).

**Six transmembrane epithelial antigen of the prostate 1 (STEAP1)** is a transmembrane protein that showed enriched expression in metastatic castration-resistant prostate cancer (mCRPC) and other solid tumors (162). An antibody-drug conjugate (ADC) targeting STEAP1 was tested in a phase I trial (NCT01283373) and showed an acceptable safety profile in patients with mCRPC (163). A phase I trial of STEAP1 and CD3 targeting bispecific-antibody is ongoing as well in patients with mCRPC (164). Bhatia V. et al. reported the study evaluating STEAP1-targeting CAR T cell therapy for mCRPC using *in vitro* and *in vivo* models. The study revealed that STEAP1 is highly expressed among mCRPC samples. Additionally, the *in vitro* cytotoxic assay was specific and strong even with minimal expression of STEAP1 on cancer cell surfaces. An *in vivo* study with NSG mice and a syngeneic mouse model demonstrated that STEAP1 CAR T cell therapy is efficacious and safe. Based on these promising results, clinical translation is underway (162).

**NKG2D** is a cell surface receptor usually expressed on cytotoxic immune cells. NKG2D is a natural receptor on the surface of natural killer cells that can recognize multiple ligands on the tumor cells. NKG2D therefore could be an efficient chimeric antigen receptor for these tumors. NKG2D ligands are present in tumor cells of various origins including colorectal cancer, ovarian carcinoma, pancreatic cancer, prostate cancer, acute lymphoblastic leukemia, lymphomas, and in a range of cancer cell lines indicating their use for both hematological and solid tumors (165, 166). NKG2D-targeting CAR T therapy was evaluated in patients with advanced solid tumors (NCT04107142 and NCT05302037).

**Ephrin type-A receptor 2 (EphA2)** is a transmembrane glycoprotein observed in epithelial cells during proliferation. EphA2 is upregulated in malignant lymphomas and EphA2-positive metastatic solid tumors, and it is regarded as a target for CAR T cell therapy (NCT05631886 and NCT05631899). A CAR T cell trial targeting epithelial cell adhesion molecule (EpCAM) is ongoing now (NCT02915445) (167). **CD22** and **CD79**, B cell membrane antigens, are targets for CAR T cell therapy in patients with CD22-positive and CD79-positive B cell malignancies given their *in vivo* results (NCT04556669 and NCT02830724) (168). **Lewis Y** is a subtype of blood group related antigens and is associated with poor prognosis in breast cancer (169). Lewis Y specific CAR T cell therapy showed efficacy in an *in vivo* trial, and a phase I trial was conducted (NCT03851146) in patients with Lewis Y antigen-expressing solid tumors. This infused CAR-T therapy displayed poor persistence in patients; however, further tuning through utilization of features of stem-like T cells in designing the CAR T cell therapy may lead to better clinical performance (170).

## 9 Discussion

### 9.1 Therapeutic limitations, challenges, and scopes for improvement

While there are positive results associated with the presented clinical trials, there remain significant therapeutic limitations. As evidenced by the presented CAR T therapy clinical trials for solid tumor treatment, a recurring theme was adverse event reporting in patients due to dose limiting toxicities (DLT). Namely, clinical trials for CAR T therapy in HER2 (NCT04650451) and PSCA(NCT02744287) were suspended due to DLTs. Another recurring challenge in trials was cytokine release syndrome (CRS), which is the most common and one of the most critical adverse events associated with CAR T therapy. Many trials, notably CAR T trials targeting HER2 (NCT04650451), Claudin 18.2 (NCT03874897), GD2 (NCT03373097), PSCA (NCT02744287), and PSMA (NCT04249947, (NCT03089203), reported that patients experienced CRS (regardless of severity). As such, the management of DLTs and CRS are pivotal issues in successful treatment and advancement of CAR T therapies for solid tumors.

CRS usually occurs within the first week of treatment, and its clinical manifestation varies from fever to severe hypotension requiring vasopressors. The mechanism of development is related to the release of inflammatory cytokines such as IFN-γ, IL-6, IM-10, macrophage inflammatory protein (MIP)1β, monocyte chemoattractant protein (MCP)-1, and GM-CSF (171, 172). Several factors may predict a higher possibility of CRS – high tumor burden, high CAR T dose, prior lymphodepletion conditioning regimen, and high peak CAR T cell counts (173). Ironically, CRS may also be associated with a positive response to CAR T cell therapy given its immune-stimulatory mechanism. Secondary hemophagocytic lymphohistocytosis/macrophage activation syndrome (HLH/MAS) originates from immune hyperactivation, which shares common mechanisms with CRS. Thus, HLH/MAS may overlap with CRS. Furthermore, serum markers in severe CRS meet the criteria of HLH/MAS.

Neurotoxicity, known as immune effector cell-associated neurotoxicity syndrome (ICANS), is also a common adverse event. It occurs in approximately 65% of patients with hematologic malignancy (174). ICANS presents with tremors, dysgraphia, aphagia, delirium, and altered mentality in early phases; ICANS can deteriorate to seizures, stupor, obtundation, and coma, sometimes associated with cerebral edema. The exact mechanism of ICANS has remained unclear, but immune stimulating cytokines such as IL-1, IL-6, and GM-CSF play roles in its development (175, 176). The inflammation caused by myeloid cells can induce capillary leaks, endothelial activation, and dysfunction of the blood-brain barrier.

Optimization of the efficacy of CAR T therapy aims to maximize immune responses with acceptable toxicity. IL-6 inhibition with tocilizumab is a widely used method to control excessive reactions; similarly, corticosteroids suppress overall immune cells (177). These two drugs are the backbone of CRS management. ITK inhibition by ibrutinib may also reduce the activity of inflammatory cytokines (178, 179).

Zarei et al. showed that the immunomodulatory drug lenalidomide enhances the effect of NKG2D CAR T cells to kill colorectal cancers *in vitro* (180). The NKG2D CAR T cells, generated from transduction of second generation NKG2D-CAR, showed significant cytotoxic activity against colorectal cancer cell lines, HCT116 and SW480, as compared to non-transduced parental T cells. Addition of lenalidomide showed dose dependent increase in the cytotoxicity and cytokine secretion by NKG2D CAR T cells. This study outcome suggests that combinational therapy utilizing NKG2D-based CAR T cells and lenalidomide has a high potential for effectively eliminating tumor cells *in vitro* and that immunomodulatory medication lenalidomide (LEN) may increase the effectiveness of CAR T cells in the treatment of solid tumors.

Additionally, the effect of natural products on the function of CAR T cells has been investigated. Huang et al. (2023) assessed the effect of gastrodin (GAS) on CAR T cells targeting interleukin-13 receptor α2 antigen (IL-13Rα2 CAR T) in the brain against glioblastoma multiforme in both *in vitro* and *in situ* glioblastoma models (181). In a transwell assay, GAS increased the migratory and destructive capacity of IL-13Rα2 CAR T cells with no effect on cytokine release. There was elevated expression of S1P1 with GAS treatment which encouraged the entry of CAR T cells into the brain and bone marrow. In the transcriptomic analysis, there was upregulation of genes such as add2 and gng8 related to skeletal migration. GAS treatment improved the mobility of IL-13Rα2 CAR T, enhancing their ability to recognize the tumor antigen of glioblastoma, indicating its potential application of CAR T for the treatment of solid tumors.

These studies show that the utilization of drugs and natural products can further improve CAR T cell function *in vitro*.

#### 9.1.1 Tumor antigen heterogeneity and loss

Unlike in hematogenic malignancies, heterogeneous expression of target antigens remains a challenge for CAR T cells in solid tumors. Target antigens may be expressed in benign tissues and are not unique in certain types of tumors. Therefore, finding tumor-specific antigens for subsequent CAR T design is a key factor in success of this therapy. Moreover, loss of antigen expression by subclonal evolution also causes treatment failure in CAR T therapy (182).

Targeting multiple antigens can help overcome the problem posed by the loss of antigen expression. For example, dual targeting of HER2 and IL13Rα2 antigen in GBM revealed an anti-tumor potential of dual-targeting CAR T cells. Blocking of the HER2 protein may facilitate selection of HER2 negative cells through an antigen escape mechanism; however, targeting IL13Rα2 made up for the negative effect perpetrated by the antigen escape mechanism (183). In the case of breast cancer, dual targeting of HER2 and MUC1 demonstrated efficacy in an *in vitro* model (184). In addition, CAR T cells that secreted EGFR-specific bi specific T cell engagers (BiTEs) for patients with glioblastoma were designed (185). These secreted BiTEs helped CAR T cells kill EGFR expressing tumor cells by recruiting bystander T cells as well as re-directing CAR T cells. In an *in vivo* model, CAR T only injection did not eliminate tumor cells completely and allowed the growth of remaining EGFR negative glioblastoma tumor cells (185). However, the injection of BiTes removed this heterogeneity. On top of increasing efficacy, the combination treatment can be activated carefully when both antigens are detected simultaneously.

One drawback in targeting multiple antigens remains targeting even more antigens expressed in normal tissue. Therefore, obtaining precise control to isolate tumor cells remains a challenge to manage on-and off-target effects. Usage of multiple CAR T cells targeting a single antigen each is another option to overcome heterogeneity (186). Co-administration of PSCA and MUC1 targeted CAR T cells showed a tumor-killing effect in an *in vivo* mice model implanted with NSCLC cells (92). In the case of cholangiocarcinoma, EGFR and CD133 revealed synergistic effects, and the patient gained sustained disease control for thirteen months (8.5 months in PR and 4.5 months in SD) (187).

#### 9.1.2 Tumor infiltration of CAR T cells

Unlike in hematologic malignancies in which CAR T cells directly contact tumor cells in the bloodstream, trafficking and infiltration into the immunosuppressive tumor microenvironment are principal issues in delivering CAR T cells to solid tumors. First, abnormal vascularization of tumors of the factors may impact tissue infiltration by reducing adhesion molecules on the endothelium (10). Therefore, several experiments attempted to overcome this barrier. Deng et al. showed that adding combretastatin A-4 phosphate (CA4P), a vascular disrupting agent, enabled increased infiltration of CAR T cells (18). Moreover, tumor cells may modulate this process by modifying multiple membrane proteins and cytokine secretion. To overcome this, a CAR T cell was designed with receptors to detect chemokines released from tumor cells (188). For example, the chemokine receptors (CCR) were expressed in engineered CAR T cells and led to effective trafficking of the engineered T cells to tumors. CCR2 – CCL2 is considered the main mechanism in intratumoral trafficking of cell therapy. GD2 and MSLN targeting CAR T cells showed increased infiltration activity by acknowledging CCR2b (189, 190). In addition, the modified CXC chemokine receptor 2 (CXCR2) in T cells engaged with CXCL1 in melanoma cells and increased migration of T cells to melanoma tissue (191). CXCR1- and CXCR2 modified CD70 engineered CAR T cells demonstrated increased intratumoral infiltration in glioblastoma in an *in vivo* model. The action of CXCL8, a chemokine related to proliferation and invasion, was neutralized by CXCR1 and CXCR2 modified CAR Ts (192). Another strategy for delivering CAR Ts was to break physical barriers by degrading matrices. Targeting fibroblast activation protein (FAP) demonstrated enhanced intratumoral delivery of CAR T cells (193). Similarly, CAR T cells producing heparinase enzyme (HPSE) also broke down the barrier and increased trafficking to target tumor tissues (194).

#### 9.1.3 Immunosuppressive tumor microenvironment

The immunosuppressive TME plays a vital role in decreased activity and the decreased tumor infiltration of CAR T cells. In addition to the hostile surrounding environment, consisting of the extracellular matrix and cancer-associated fibroblasts, immune suppressive cells such as regulatory T cells (Treg), TRIF-related adaptor molecule (TRAM), and myeloid-derived suppressor cells (MDSC) interfere with the antitumor activity of CAR T cells (195). Hypoxic conditions and immunosuppressive cytokines such as TGF-β can hamper the treatment. Reversing the suppressive cytokine and their respective pathways is a strategy to overcome these challenges.

Engineered TGF-β dominant-negative receptor (DNR) has inhibitory effects on TGF-β signal. These effects block the transformation of primitive T cells into Treg. A CAR T therapy targeting PSMA with TGF-β DNR is in an ongoing phase I clinical trial (NCT0089203). The trial looks to show the reversible effects to the suppressive environment. PD-1/PD-L1 and FAS/Fas ligand pathways also have similar inhibitory effects in immune boosting reactions. Such results were demonstrated as CAR T cells engineered with PD1 DNR expression or with FAS DNR expression had increased activity in *in vivo* models (95, 96).

Enhancing immunostimulatory cytokines remains another option in battling the immunosuppressive tumor microenvironment. IL-12 secreting CAR T cells highlighted their tumor killing effect by reducing Treg activity and empowering IFNγ secretion (31, 196). IL-18 has a similar role in its proinflammatory action in CAR T therapy (197). Suppressing immune-regulatory cells like myeloid-derived suppressor cells (MDSCs) can be an option to overcome resistance - modulating reactive oxygen species with all-trans retinoic acid (ATRA) can reduce the activity of MDSCs. Engineered NK cells targeting GD2 act on the impaired activity of MDSCs when co-administered with CD2 targeted CAR T cells in a neuroblastoma model (198).

Additionally, colony-stimulating factor 1 receptor (CSF1R) and granulocyte macrophage-colony stimulating factor (GM-CSF) can regulate the activity of tumor-associated macrophages (TAM). Hence, targeting CSF1R and GM-CSF may facilitate an anti-tumor effect of CAR T cell therapy (199, 200).

### 9.2 CRISPR gene editing for boosted CAR T cell activity

Some gene mutations were identified as enhancers to autologous CAR T cell therapy through CRISPR screening. For example, the deletion of TCR encoding genes and inactivation of PD-1 by CRISPR/Cas9 methods caused an increase in immune activity (19). In addition, the knock-down of Tet methylcytosine dioxygenase 2 (TET2) encoding genes led to the improved efficacy of CD19-based therapy (20). CRISPR knock-out screens of RASA2, which is a RAS GTPase-activating protein, in T cells led to increased T cell activation and led to more persistent cancer cell killing (201). Similarly, an *in vivo* pooled CRISPR-Cas9 screening approach targeting REGNASE-1 showed that T cells can be reprogrammed to long-lived effector cells with better persistence in tumors as compared to control T cells. Further CRISPR-Cas9 screens showed that BATF, the key target of REGNASE-1, loss led to the greater accumulation of REGNASE-1 deficient T cells (202).

Additionally, deletion of DNA methyltransferase 3 alpha (DNMT3A) indicated the enhanced immunogenicity and inhibition of T cell exhaustion (203). The recent study by Garcia J. et al. demonstrated that the other fusion mutation in caspase recruitment domain-containing protein (CARD11) and Phosphoinositide-3-Kinase Regulatory Subunit 3 (PIK3R3) improved tumor killing effect by enhanced signaling of CARD11–BCL10–MALT1 complex (204). Therefore, the exploration of various genetic mutations in T cell biology can be a promising solution for improvement and overcoming resistance. In the context of engineering CAR T cells, the overexpression of canonical AP-1 factor c-Jun has enhanced T cell expansion potential, increased functional capacity, and improved anti-tumor response in multiple *in vivo* mouse tumor models (205).

In November 2023, the FDA announced an investigation regarding the risk of T cell malignancies, including CAR positive lymphomas, for those who received BCMA or CD19 directed CAR T therapies. The recent report by Ghilardi et al. indicated that three cases of T cell lymphoma were observed after anti-CD19 CAR T therapy for non-Hodgkin B cell lymphoma was administered (206). According to this report, further studies among patients treated at the University of Pennsylvania showed that 3.6% (16 of 449 patients) reported a secondary malignancy – one case was identified as T cell lymphoma with a very low incidence. In addition, Garcia et al. discussed the concerns associated with T cell malignancies in those administered these CAR T cell therapies (204). Their study, and namely their experimental data derived from a longer term follow up in murine studies, did not suggest that there was an increased risk of developing secondary T cell malignancies when fusion genes were inserted into CAR T cells. Typically, these insertions are one of the naturally occurring mutations in human T cell cancers. Instead, Garcia et al. concluded that pre-existing genetic features could contribute to secondary sporadic malignancies. Moving forward, the field will need to continue address these concerns and adhere to a more cautious approach until more evidence is gathered.

### 9.3 Conclusion and outlook

CAR T therapy is proving to be a viable option in cancer treatment, especially with the advancements in CAR engineering. The areas of concern, namely antigen heterogeneity, the immunosuppressive tumor microenvironment, and tumor infiltration, have been identified. Focused research in these areas, as well as in expanded areas of CAR engineering and antigen discovery, has progressed. One of the prevailing problems that remains is that often CAR Ts do not reach target antigens in solid tumors in sufficient numbers because of their destruction by lymphatic system or by the tumor microenvironment. To overcome these problems, a nanoparticle-based packaging and delivery system is being examined. A variety of nanotechnologies, including hydrogel, nanoparticle conjugation, transient CAR expression in T cells through RNA delivery, and others are being explored (207). These advances and further engineering of CARs looks to further the ability of CAR T cell therapy to be used as a therapeutic approach for solid tumors.
